# Patient-Derived Cancer Organoids as Predictors of Treatment Response

**DOI:** 10.3389/fonc.2021.641980

**Published:** 2021-03-18

**Authors:** Maikel Verduin, Ann Hoeben, Dirk De Ruysscher, Marc Vooijs

**Affiliations:** ^1^ Department of Radiation Oncology (MAASTRO), GROW School for Oncology and Developmental Biology, Maastricht University Medical Center+, Maastricht, Netherlands; ^2^ Department of Medical Oncology, GROW School for Oncology and Developmental Biology, Maastricht University Medical Center+, Maastricht, Netherlands

**Keywords:** organoids, precision medicine, treatment response, treatment prediction, cancer, patient-derived

## Abstract

Patient-derived cancer organoids have taken a prominent role in pre-clinical and translational research and have been generated for most common solid tumors. Cancer organoids have been shown to retain key genetic and phenotypic characteristics of their tissue of origin, tumor subtype and maintain intratumoral heterogeneity and therefore have the potential to be used as predictors for individualized treatment response. In this review, we highlight studies that have used cancer organoids to compare the efficacy of standard-of-care and targeted combination treatments with clinical patient response. Furthermore, we review studies using cancer organoids to identify new anti-cancer treatments using drug screening. Finally, we discuss the current limitations and improvements needed to understand the full potential of cancer organoids as avatars for clinical management of cancer therapy.

## Introduction

Cancer remains one of the leading causes of death in the 21st century. Despite enormous progress in the identification of mechanisms of tumor progression and treatment resistance and the development of new tumor targeted treatments many patients do not get cured. The main challenge remains to achieve accurate patient selection. Tumor biopsies used for clinical diagnostics do not capture the extensive intratumoral heterogeneity that masks emergent tumor clones with intrinsic or acquired resistance. This can result in patients receiving suboptimal treatment, or overtreatment leading to long-lasting harmful side-effects. The development of specific biomarkers and predictive model systems are therefore essential to personalized treatment leading to more durable responses with fewer side effects.

In the last decade, translational cancer research has witnessed a revolution with the development of methods that enable the reproducible derivation, maintenance and biobanking of primary human normal and cancer tissues. These primary cell cultures, called organoids, are three dimensional stem-cell derived cultures that support the propagation of phenotypic, genetic and transcriptomic characteristics from the original tissue and retain the self-renewal properties of stem cells and their ability to undergo multilineage differentiation indefinitely. This revolution took off after the development of ‘mini-guts’ from Lgr5+ intestinal stem cells which replicate the dynamic proliferation and differentiation of the intestinal crypt epithelium in culture (1) and thereafter from colorectal cancer biopsies to derive colorectal cancer (CRC) organoids (2). Importantly cancer organoids have been shown to retain intratumoral heterogeneity and tumor clonal hierarchy; a major limiting factor in treatment effectiveness and recurrence (3). Excellent comprehensive reviews on the derivation and characterization of organoid systems can be found here and will not be further elaborated on in this review (4). To date cancer organoids have been developed from many cancer types and have been shown to maintain a stable genetic and phenotypic representation of the original tumor in culture compared to classic 2D monolayer cell cultures. Like patient-derived xenograft (PDX), cancer organoids also have maintained intratumor heterogeneity but are less expensive and labor-intensive and reduce laboratory animal usage. On the other hand, PDX models include the tumor micro-environment and *in vivo* drug metabolism which are currently not accounted for in cancer organoids (5).

Whether cancer organoids can also function as avatars for prospective target identification and treatment selection at the patient level is not yet known. If so, cancer organoids could have a profound impact on individualized cancer treatment. In this review, we describe studies that have addressed whether cancer organoids are predictors for clinical response.

## Materials and Methods

A literature search was conducted using publicly available databases (PubMed, MEDLINE, and Web of Science) up to October 2020. For the purpose of this review, cancer organoids were defined as stem-cell derived three-dimensional cell culture models derived from primary patient-derived solid tumors using a basement membrane extract (BME). Articles describing patient-derived-xenograft (PDX)-derived cancer organoids, iPSC based models or three-dimensional cell culture systems lacking a BME (i.e. slice cultures or tumor-on-a-chip approaches) were excluded. Search keywords included [ORGANOID] or [TUMOROID] and, [CANCER] and [TREATMENT]. Only articles in which cancer organoids were subjected to treatment and correlated to patient outcome or potential biomarkers were included. Establishment, characterization and comparison of cancer organoids towards the parental tumor have already been previously described and fall outside the scope of this review (6, 7).

## Results

A total of 60 studies were included in this review. For almost all papers the cancer organoids used were (previously) compared on a genetic and phenotypic level to the parental tumor in order to validate the model. Study characteristics are summarized in **Table 1**.

**Table 1:** Summary of study characteristics and main results.

Study	Cancer type	Organoid establishment success rate (%)	# of organoid lines used for treatment experiments	Multiple organoid lines per patient	Matched healthy tissue organoids		Standard-of-care testing	Clinical comparison	Drug screen	Outcomes regarding prediction of clinical treatment response
**Gastro-intestinal cancer**										
van de Wetering M. et al. (2)	Colorectal cancer	90%	19							Drug screen on organoids. No clinical comparison.
Koppens M. et al. (8)	Colon cancer	not reported	2							Efficacy of EZH2 inhibition in organoids. No clinical comparison.
Verissimo CS. et al. (9)	RAS mutant colorectal cancer	n.a.	2							Organoid response towards EGFR-RAS-ERK targeting in relation to KRAS mutation status. No clinical comparison.
Buzzelli JN. et al. (10)	Colorectal cancer liver metastases	76.5%	3							Efficacy of standard-of-care chemotherapy in organoids. No clinical comparison.
Vlachogiannis G. et al. (11)	Metastatic gastrointestinal tumors	not reported	21							Sensitivity 100%, specificity 93%, PPV 88%, NPV 100% for organoids in forecasting clinical response to targeted agents or chemotherapeutics.
Gao M. et al. (12)	Gastric cancer	not reported	2 (from 1 patient)							Testing of standard-of-care chemotherapeutics. Descriptive clinical comparison (N=1) showed lowest IC50 value for 5-FU (out of 4 chemotherapeutics tested) and clinical complete response after 5-FU/RTx treatment. No testing for contribution of RTx to clinical effect.
Li X. et al. (13)	Esophageal adenocarcinoma	31%	9							Drug screen on organoids. Descriptive comparison showing lack of chemotherapy sensitivity in most organoid cultures which resembled the poor clinical response observed.
Yan HHN. et al. (14)	Gastric cancer	50% (cancer), 100% (healthy)	9 (from 7 patients)							Drug screen on organoids. Descriptive comparison showing lack of organoid response to 5-FU in a patient that showed progressive disease upon 5-FU. Two other patients showed a clinical response to 5-FU/cisplatin which was resembled in the organoids.
Votanopoulos KI. et al. (15)	Appendiceal cancer	75%	9							Chemosensitivity testing of organoids. No clinical comparison.
Li J. et al. (16)	Gastric cancer (ascites-derived)	92%	7							Drug screening of organoids. No clinical comparison.
Schumacher D. et al. (17)	Colorectal cancer	not reported	38							Efficacy of EGFR-targeted therapy and its downstream targets (MEK and mTOR) in relation to KRAS mutation status in organoids. No clinical comparison.
Seidlitz T. et al. (18)	Gastric cancer	not reported	4							Drug testing of organoids (targeting HER2, c-KIT or CDK4/6). No clinical comparison.
Ubink I. et al. (19)	Colorectal peritoneal metastases	Not reported	5							Sensitivity to HIPEC chemotherapy and efficacy of addition of ATR inhibitor. No clinical comparison.
Pasch CA. et al. (20)	Multiple types of cancer (treatment only on (m)CRC)	76%	5							Descriptive clinical comparison (N=1). Clinical response to FOLFOX in a patient of which the organoid showed an intermediate response towards 5-FU/oxaliplatin treatment.
Steele NG. et al. (21)	Gastric cancer	not reported	6							Drug screening of organoids. Descriptive clinical comparison (N=2) showing a similar response in the organoid for one patient but not in the other.
Ganesh K. et al. (22)	Rectal cancer	77%	21							Drug screening of organoids. Clinical comparison for chemotherapy (N=7) showing a correlation of AUC for both 5-FU and FOLFOX with progression-free survival of the corresponding patient (r=0.86, p=0.024). Descriptive comparison of radiosensitivity (N=7) showing organoid responds corresponds with clinical radiotherapy response.
Ooft SN. et al. (23)	Metastatic colorectal cancer	63%	Varies per treatment							Prediction of response to irinotecan monotherapy (N=10): accuracy of classifier 80%. Prediction of response to 5-FU/irinotecan combination therapy (N=12): 83.3% correctly classified. Prediction of response to 5-FU-oxaliplatin (N=16): no correlation with clinical response.
Costales-Carrera A. et al. (24)	Colon cancer	not reported	3							Efficacy of plocabulin in organoids. No clinical comparison.
Yao Y. et al. (25)	Locally advanced rectal cancer	85.7%	80							High correlation between organoid response and clinical outcomes for prediction of neoadjuvant chemoradiation efficacy: AUC 88.20% (76.46-98.67%), accuracy 84.43% (72.40-93.75%), sensitivity 78.01% (55.56-95%), specificity 91.97% (77.78-100%).
Narasimhan V. et al. (26)	Colorectal peritoneal metastases	68%	15							Drug screening of organoids. Descriptive clinical comparison (N=3) in which drug treatment was selected based on organoid sensitivity which was successful for 1 patient.
Zerp SF. et al. (27)	Colorectal cancer	not reported	3							Efficacy of APG-880 as a radiosensitizer in organoids. No clinical comparison.
Derouet MF. et al. (28)	Esophageal adenocarcinoma	57.2%	16							Descriptive clinical comparison (N=4) showing an overlap between the organoid and tumor response.
Arena S. et al. (29)	Colorectal cancer	not reported	5							Drug testing on organoids. Descriptive clinical comparison (N=3) which corresponded with organoid sensitivity.
**Abdominal (non-GI tract) cancer**										
Huang L. et al. (30)	Pancreatic cancer	not reported	5							Drug screen of organoids. No clinical comparison.
Broutier L. et al. (31)	Liver cancer	44%	6							Drug sensitivity testing of organoids. No clinical comparison.
Nuciforo S. et al. (32)	Hepatocellular carcinoma	26%	12							Efficacy of sorafenib on organoids. No clinical comparison.
Tiriac ML. 2018 (33)	Pancreatic cancer	75%	66							Descriptive comparison of organoid response towards clinical response. For one patient retrospective clinical data paralleled the chemosensitivity profile of the organoid.
Li L. et al. (34)	Liver cancer	not reported	27 (from 5 patients)							Drug screen of organoids. No clinical comparison.
Hennig A. et al. (35)	Pancreatic cancer	71%	10							Efficacy of standard-of-care chemotherapy stratified for KRT81 status. No clinical comparison.
Bian B. et al. (36)	Pancreatic cancer	not reported	24							Efficacy of BET-inhibitor treatment on organoids. No clinical comparison.
Driehuis E. et al. (37)	Pancreatic cancer	62%	24							Drug screen on organoids. Descriptive comparison towards clinical response (N=4) showing an overall correlation between organoid and clinical response.
Ponz-Sarvise M. et al. (38)	Pancreatic cancer	not reported	2							Drug sensitivity testing of organoids. No clinical comparison.
Castven D. et al. (39)	Liver cancer	11%	5							Testing efficacy of targeted agents based on mutational variants in organoids. No clinical comparison.
Sharick JT. et al. (40)	Pancreatic and Breast cancer	64% (for pancreatic cancer), 54% (for breast cancer)	7 (pancreas), 11 (breast)							Using metabolic heterogeneity to predict treatment response in pancreatic cancer organoids (N=7). Three patients were classified as predicted non-responders and all showed tumor recurrence within one year whereas four patients that were classified as predicted responders all remained free of tumor recurrence for more than one year.
Seppälä TT. et al. (41)	Pancreatic cancer	77%	13							Pharmacotyping of organoids. No clinical comparison.
Saltsman J. et al. (42)	Hepatoblastoma	not reported	1							Drug testing on normal liver and tumor organoid from one patient. No clinical comparison.
Liu J. et al. (24)	Liver cancer	not reported	4							Effect of co-culture system with cancer-associated fibroblasts on drug sensitivity in organoids. No clinical comparison.
**Urogenital and gynecological cancer**										
Gao D. et al., 2014 (43)	Metastatic prostate cancer or CTCs	15-20%	6							Sensitivity to androgen receptor and PI3K inhibitors in organoids. No clinical comparison.
Girda E. et al. (44)	Endometrial cancer	100%	14 (varies per drug)							Drug testing on organoids. No clinical comparison.
Lee SH. et al. (45)	Bladder cancer	70%	11							Drug screen of organoids and comparison to in vivo (mice) response. No clinical comparison.
Puca L. et al. (46)	Prostate cancer	16%	6							Drug screening on organoids. No clinical comparison.
Kopper O. et al. (47)	Ovarian cancer	65%	21							Descriptive clinical comparison: organoids derived from clinical resistant recurrent disease were more resistant compared to the clinically sensitive primary disease counterpart (N=1). Drug screen of organoids and comparison to in vivo (mice) response.
Boretto M. et al. (48)	Endometrial cancer	20%	5							Drug response to standard-of-care chemotherapeutics. No clinical comparison.
Mullenders J. et al. (49)	Bladder cancer	57.9%	3							Drug response to standard-of-care chemotherapeutics. No clinical comparison.
Calandrini C. et al. (50)	Childhood kidney cancer	100% for healthy tissue, 75% for Wilms tumor, 100% for MRTK, 75% for RCC. Unsuccessful for rare kidney tumor types	4							Drug screen of cancer and healthy tissue organoids. No clinical comparison.
de Witte C.J. et al. (51)	Ovarian cancer	not reported	36							Drug screening on organoids. Organoid drug response to carboplatin+paclitaxel treatment showed significant correlation with clinical response (N=7, P<0.01). PDOs generated at interval debulking recapitulated the clinical response to first-line carboplatin and paclitaxel combination treatment for histopathological (p = 5.821e 05), biochemical (p = 0.0004), and radiological (p = 0.0092) outcomes.
**Central nervous system cancer**										
Hubert CG. et al. (52)	Glioblastoma	not reported	1							Identification of radioresistant cells in organoids. No clinical comparison.
Saengwimol D. et al. (53)	Retinoblastoma	83%	1							Effects of standard-of-care chemotherapeutics. No clinical comparison.
Scognamiglio G. et al. (54)	Chordoma	not reported	3							Efficacy study of nivolumab. No clinical comparison.
Loong HF. et al. (55)	Glioblastoma	n.a.	1							Prospective identification of everolimus as treatment option using organoids showing subsequent partial clinical response.
Chadwick M. et al. (56)	Glioblastoma	not reported	4							Drug screen on organoids. No clinical comparison.
**Breast cancer**										
Sachs N. et al. (57)	Breast cancer	>80%	28							Drug screening of organoids and comparison to in vivo response in mice. No clinical comparison.
Li X. et al. (58)	Breast cancer	n.a.	1							Case-report for drug screening on organoids. No clinical comparison.
**Pulmonary cancer**										
Sachs N. 2019 (59)	NSCLC	28%	4							Response to multiple chemotherapeutics and TKI’s. No clinical comparison.
Kim M. et al. (60)	Lung cancer	87%	5							Response to docetaxel, olaparib, erlotinib and crizotinib. No clinical comparison.
Chen J. et al. (61)	NSCLC	not reported	7							Response to chemotherapeutics and targeted agents in organoids. No clinical comparison.
Li Z. et al. (62)	NSCLC	80%	12							Drug screen on organoids. No clinical comparison.
**Head-and-neck cancer**										
Tanaka N. et al. (63)	Head-and-neck cancer	37.2%	4							Response to cisplatin and docetaxel. No clinical comparison.
Driehuis E. et al. (64)	HNSCC	65%	13							Descriptive comparison of response to radiotherapy (N=7). Organoid response for 6 patients was similar to the observed clinical response. Healthy organoids were not subjected to treatment.
Driehuis E. et al. (65)	HNSCC	n.a.	8							Efficacy of EGFR-targeted photodynamic therapy. No clinical comparison.

### Gastro-intestinal (GI) Cancer

In 2015 the first colorectal cancer (CRC) organoid biobank was established and used for drug screening. Based on these drug screens, gene-drug interactions could be studied to identify potential biomarkers and study the molecular basis for drug response to both new therapeutic agents as well as current standard-of-care chemotherapeutics (2).

Esophageal adenocarcinoma organoids were subjected to standard-of-care chemotherapy (5-FU, epirubicin and cisplatin). All but one of the patients used for organoid derivation showed poor clinical response to chemotherapy which was recapitulated in the corresponding cancer organoids, though organoids from the patient that showed clinical response were not available for drug testing (13). This overlap between organoid and tumor response for four patients towards chemotherapeutics (cisplatin, paclitaxel, 5-FU, epirubicin and irinotecan) was also observed in another study (28).

A similar approach was taken using gastric cancer organoids. In one study, gastric cancer organoids derived from one patient at pre-treatment were sensitive to standard-of-care chemotherapy (5-fluorouracil (5-FU), cisplatin, oxaliplatin and irinotecan) which reflected the complete pathologic response in the patient after chemoradiation, albeit the contribution of radiotherapy to the clinical response was not investigated (12). In another study conflicting results for combined treatment with 5-FU, oxaliplatin and epirubicin were obtained. From the seven gastric cancer patients included in the study, correlation of treatment sensitivity (5-FU, oxaliplatin and epirubicin combination treatment) with clinical response could only be made for two patients. Only for one of these patients the organoid response matched the clinical response (21).

Ascites-derived gastric cancer organoids showed a heterogeneous response towards standard-of-care chemotherapeutics (oxaliplatin, 5-FU, cisplatin, docetaxel, irinotecan, epirubicin and paclitaxel) between patients, similar to the mixed clinical responses seen in patients with peritoneal metastases, though no direct clinical comparison could be made (16).

CRC organoids were used to identify patients that benefit from the PARP inhibitor olaparib and cross sensitivity to oxaliplatin, which causes PARP-dependent DNA damage repair. In two patients that responded to oxaliplatin the organoids were sensitive to both olaparib and oxaliplatin. In another patient that responded, the organoids showed resistance to both treatments. Notably the organoids from this patient were highly responsive towards panitumumab which was also part of the clinical treatment and might have been a main factor in the clinical response and explain the discrepancy between organoid and clinical response (29).

The TUMOROID study used organoids derived from metastatic CRC and correlated the organoid treatment response towards the corresponding clinical response. Organoids were able to predict the response to irinotecan-based therapies in more than 80% of patients without misclassifying patients who responded to the treatment. This predictive value was not identified for 5-FU and oxaliplatin combined treatment. A possible explanation for this could be that the tumor micro-environment (stromal and immune cells), not present in organoids, might influence the efficacy of one treatment more than the other (23). In another study, organoids from one metastatic CRC patient showed an intermediate response towards 5-FU and oxaliplatin combination treatment which mimicked the clinical response observed after re-treatment with FOLFOX (20).

Treatment sensitivity analysis on peritoneal CRC metastasis organoids could not separate patients with clinical partial response from those with progressive disease after FOLFOX. However, most of the patients in this study received pre-operative chemotherapy which may have led to selection of chemo-resistant subclones. Two patients did not receive oxaliplatin-based therapies and showed the highest sensitivity towards the treatment *in vitro*. Furthermore, two treatment-refractory patients were treated with a drug that was selected based on the drug sensitivity observed in their corresponding organoids. One patient received vandetanib (pan-tyrosine-kinase inhibitor) which strongly reduced organoid viability, however no clinical response was observed. For another patient, gemcitabine showed an initial partial clinical response but after two additional months of treatment, disease progression was again observed (26). Peritoneal CRC-metastases organoids were also treated for efficacy towards mitomycin C and oxaliplatin (commonly used in HIPEC; intra-peritoneal chemotherapy treatment) and showed a general resistance, corresponding with the high recurrence rates observed in clinic (19).

Organoids from metastatic GI cancers also predicted sensitivity towards cetuximab (anti-EGFR monoclonal antibody), and reflected clinical resistance in a patient who, based on molecular markers (EGFR amplification and KRAS wild-type), was expected to respond to the treatment (11).

Rectal cancer organoids were exposed to standard-of-care chemotherapy (5-FU alone or FOLFOX (5-FU with leucovortin and oxaliplatin)) or radiotherapy (single dose, 0-8Gy). A high correlation (r=0.86) for 5-FU or FOLFOX was observed when compared to the progression-free survival (PFS) of the corresponding seven patients. For radiotherapy, organoids that showed resistance to radiotherapy were derived from previously irradiated tumors or from tumors that showed no to minimal clinical response. On the other hand, more radiosensitive organoids were derived from patients who had a minimal 50% reduction in tumor circumference endoscopically or a near-complete or a clinical complete response following radiotherapy (22). Additionally organoids (N=80) treated with neoadjuvant chemoradiation (5-FU and irinotecan) also reported promising predictive value for clinical response to neoadjuvant chemoradiation (sensitivity 78.01%, specificity 91.97%) (25).

As a proof-of-concept, the influence of KRAS mutation-mediated resistance to EGFR-targeted therapy using cetuximab was tested in rectal cancer organoids. In accordance with clinical trial data, KRAS-mutated organoids showed more resistance towards cetuximab compared to the KRAS wild-type organoids (22). These findings were also confirmed in an independent rectal cancer organoid biobank (66) and for combined EGFR and MEK inhibition in CRC organoids (9).

Multiple studies have used organoids to screen for efficacious targeted agents based on genomic targetable variants present in the organoids. This approach was taken using gastric cancer organoid biobanks, CRC cancer organoids and in esophageal cancer organoids in which drug screening approaches identified patient subsets with potential vulnerability to new targeted agents (8, 13, 14, 17, 18, 24, 67).

Finally, organoids were used to improve current treatments. CRC organoids showed an enhanced response to radiotherapy when organoids were simultaneously exposed to radiosensitizer APG-880 (27). Peritoneal CRC-metastases organoids were used to optimize HIPEC treatment. Because mitomycin C (used in HIPEC) mainly induces interstrand crosslinks which activates ATR, the addition of ATR inhibitors to mitomycin C improved treatment efficacy on cancer organoids, identifying a potential new clinical strategy (19).

### Hepatobiliary Tract and Pancreatic Cancer

Pancreatic cancer is one of the most lethal malignancies as it is often diagnosed in an advanced stage, has a high recurrence rate and only a minor survival benefit can be achieved with systemic therapy. This resistance to gemcitabine was also observed in pancreatic cancer organoids, though no clinical correlation could be made (30).

An organoid-derived pharmaco-transcriptomic signature was developed to predict drug sensitivity to gemcitabine monotherapy but it did not predict response in patients receiving a combination treatment with other chemotherapeutics (33). Similar correlation between clinical response and gemcitabine sensitivity in pancreatic cancer organoids were found by others as well (37). Another study also showed the feasibility of pharmacotyping pancreatic cancer organoids in a timely manner to guide postoperative chemotherapeutic selection (41).

Pancreatic cancer organoid cultures derived from multiple different metastatic sites from the same patient showed a differential sensitivity towards 5-FU, but not towards the other chemotherapeutics tested. This suggests the existence of cancer subclones that differ between metastatic sites which are maintained in their respective cancer organoids (33).

Optical metabolic imaging (OMI) was used to assess treatment response in pancreatic cancer organoids and classify patients. OMI is a high-resolution fluorescence microscopy technique that quantifies the metabolic state of individual cells. This technique measures drug response in heterogeneous 3D populations faster than more traditional methods, as changes in cell metabolism precede changes in cell viability (40). Using OMI on cancer organoids, three patients were classified as predicted non-responders and all showed tumor recurrence within one year whereas four patients that were classified as predicted responders all remained free of tumor recurrence for more than one year (40).

Novel approaches for pancreatic cancer organoids include the co-culture with cancer-associated fibroblasts (CAFs) and using matched pancreatic ductal organoids. CAFs were shown to increase treatment resistance which shows the importance of tumor micro-environmental aspects on treatment efficacy (68). Using matched pancreatic ductal organoids and pancreatic cancer organoids the lack of therapeutic response of dual MEK-AKT inhibition observed in a clinical phase II trial (69) was investigated. These findings recapitulated in human and murine organoids a tumor cell specific negative feedback loop causing upregulation of ERBB2, which was not observed in normal pancreatic ductal organoids. This provides a rationale for combining dual MEK-AKT inhibition with ERBB2 blockade with a high therapeutic ratio (38).

Liver cancer organoids can be derived from liver cancer subtypes (hepatocellular carcinoma (HCC) cholangiocarcinoma (CC) or mixed type (HCC/CC)). Overall differential sensitivity was found between organoids from different patients to chemotherapies (gemcitabine) and targeted therapies (taselisib, AZD8931, SCH772984 and dasatanib or sorafenib) (31, 32), though no clinical correlations could be made.

Liver cancer organoids (n=7) were also subjected to targeted therapies (KRAS, MET and KIT targeting). Sensitivity to these treatments however did not fully correlate with the presence of these driver mutations (39). In a more comprehensive study, liver cancer organoids from different tumor regions from the same patient were developed (n=5, 27 organoid lines) and tested for their sensitivity to conventional therapies and drug screening. Most interestingly, pan-effective drugs were identified that could uniformly kill many organoid lines whereas other drugs were only moderately sensitive in a few organoids from the same patient. This study highlights the intratumoral heterogeneity and differential sensitivity towards treatment within one patient and that a single patient organoid may not be sufficient to predict treatment outcome (34).

Cancer organoids were also derived from pediatric hepatoblastoma. For one patient drug testing was performed on the matched normal liver and tumor organoids. This screen identified one drug (JQ1) to have an increased efficacy on tumor organoids compared to normal organoids whereas standard-of-care cisplatin had not differential effect (42). Matched normal liver tissue organoids were also developed by others, providing opportunities to test normal tissue toxicity (32).

### Urogenital and Gynecological Cancer

Bladder cancer organoids were tested for sensitivity to chemotherapeutics (epirubicin, mitomycin C, gemcitabine, vincristine, doxorubicin or cisplatin), though no correlations could be made with patient response (49). Another study took a drug screening approach using bladder cancer organoids and observed strong, but variable responses. For example, in some organoids from patients with FGFR3 activating mutations, MEK/ERK inhibition was effective but not in all. Correlations were seen between more aggressive clinical phenotypes (metastasis and recurrence) and treatment resistance to a wide range of drugs in organoids (45).

A biobank (>50 organoid lines) of pediatric kidney cancer organoids was used to test treatment sensitivity towards standard-of-care chemotherapy (neoadjuvant actinomycin D (ACT-D) and vincristine; adjuvant doxorubicin and/or etoposide) on a specific subset of pediatric kidney cancers (Wilms tumor). Organoids derived from patients that received neoadjuvant chemotherapy were less sensitive to vincristine than those from patients not receiving prior chemotherapy. This suggests that resistance already develops *in vivo* and is maintained in cancer organoids. Furthermore, high-throughput drug screens in Wilms tumor organoids identified MEK and HDAC inhibitors as novel candidate interventions. Importantly, matched normal kidney organoids used in this study as well were equally sensitivity to romidepsin (HDAC inhibitor) and MEK inhibition compared to the tumor organoids. For pabinostat (pan-HDAC inhibitor) a significant increased sensitivity was observed in the tumor organoids compared to the normal kidney organoids making this the most interesting for clinical use. Using this approach, this study sets an example of using matched healthy and tumor organoids to identify treatments with the best therapeutic ratio, considering both tumor efficacy and normal tissue toxicity (50).

Prostate cancer organoids were developed from neuro-endocrine prostate cancer (NEPC) and used for screening of cytotoxic drugs and identified alisertib (aurora A-kinase inhibitor). Organoids from two patients enrolled in a phase 2 clinical trial of alisertib mimicked the clinical response of the patients (one responder, one non-responder), supporting the potential clinical relevance of NEPC organoids as a predictive platform (46, 70).

Castrate-resistant prostate cancer (CRPC)-derived organoid lines (n=7) could successfully be established from biopsies and circulating tumor cells from all subtypes. A very limited drug study showed a response towards androgen suppression therapy (enzalutamide) in an organoid with androgen receptor amplification. This organoid line also showed a response to everolimus (mTOR inhibitor) and buparlisib (PI3K inhibitor) correlating with mutations in PTEN and PIK3R1 (43).

Endometrial cancer organoids (EC-O) representing early hyperplastic endometrium (n=13) and different stages and grades were derived (n=16) and exposed to standard-of-care treatment (paclitaxel, 5-FU, carboplatin, doxorubicin) and everolimus. These studies showed differential response between organoids from different patients (48). Similar patient-specific responses were observed in another study with low- and high-grade EC-O (n=15) (44). Additionally, organoids from a patient with uterine carcinosarcoma and a patient with endometrial adenocarcinoma were used for drug screening. In both patients PI3K-inhibitors (buparlisib) were most effective, consistent with PIK3CA mutation present and strongly interacted with HDAC inhibitors as the most potent combinations treatment for both cancer organoids (67). For all three studies, no clinical comparison could be made.

Ovarian cancer organoids (OC-O) were successfully developed from multiple stages and subtypes (56 organoid lines). Standard-of-care drugs (platinum/taxanes) as well as targeted agents (PIK3K/AKT/mTOR inhibitors or PARP inhibitors) were used to test treatment sensitivity of OC-O. Unsupervised hierarchical clustering based upon platinum/taxane sensitivity could distinguish chemosensitive high-grade serous (HGS) organoids and chemoresistant non-HGS organoids which corresponded to the clinical findings. For one patient, HGS organoids clustered with the resistant group, corresponding to its clinically chemo-resistant phenotype and status as recurrent disease. The pre-treated counterpart of this patient did cluster with the chemo-sensitive group, correlating with clinical behavior (47). Another study also developed OC-O from different subtypes and found significant correlations between organoid response towards carboplatin and paclitaxel combination treatment and clinical response (N=7, p<0.01). During follow-up, organoid drug response did not correlate with 6-month progression-free survival. However, organoids derived from the patient with the shortest overall survival was least responsive. Interestingly, for a subset of patients, organoids were derived from multiple biopsies from the same tumor. This identified a differential response in monotreatment between different cancer lesions of seven patients in 31% of the cases, with at least one-mismatch for every drug tested. This emphasizes that intratumor heterogeneity may not be captured by a single biopsy indicating the importance of using multiple biopsies from different locations to predict sensitivity (51).

### Central Nervous System Cancer

As a proof-of-concept, organoids from one GBM patient, progressive after standard-of-care treatment, was used to identify potential drug candidates based on genomic alterations. This identified everolimus to be a potential therapeutic agent which correlated with a partial clinical response in this case-study (55). Cancer organoids derived from GBM patients were also used to test drug sensitivity to both standard-of-care chemotherapy (temozolomide) and molecular targeted agents towards mTOR, PI3K or DNA damage response. Differential response towards monotherapy as well as combined treatments with temozolomide and targeted agents was observed between organoids from different patients (56).

Chordoma organoids, a rare spinal cancer, were established that retained PD1 positive CD8 T-cells and were used to predict response towards nivolumab (PD-L1 blockade). A dose-dependent effect was observed in both PD-L1 positive and PD-L1 negative patients which corresponds with previous findings that low expression of PD-L1 can still lead to responses towards PD-L1 blockade (i.e. the approval of PD-L1 inhibitor pembrolizumab in NSCLC starting at 1% PD-L1 positivity). This study shows that this treatment response can potentially be predicted in cancer organoids, regardless of PD-L1 status though no correlation towards individual patient response could be made (54).

Multiple organoid lines of retinoblastoma were developed and tested for response to standard-of-care chemotherapy (melphalan, topotecan and methotrexate) which showed a similar response towards tumor cells in advanced disease in clinical practice, but here no direct comparison to the patient response was made (53).

### Breast Cancer

Breast cancer is one of the most prevalent types of cancer, comprising over 20 different subtypes. To include all these subtypes, a large (>100 organoid lines) breast cancer organoid biobank was established. For 12 patients with metastatic breast cancer a comparison with patient outcome was made. In this subset, the *in vitro* response to tamoxifen (estrogen receptor antagonist) matched that of the patients, showing their potential as treatment predictors. This biobank was used for high-throughput drug screening in which most, but not all, organoids responded to treatment as predicted from their mutations. Some organoid lines were insensitive to HER2 targeting despite HER2 overexpression which emphasizes the value of functional *in vitro* drug tests using cancer organoids (57).

In another proof-of-concept study breast cancer organoids were derived from one patient and drug screening identified fulvestrant (estrogen receptor antagonist) as the most optimal treatment for this patient whereas based on genetic analysis (PTEN mutant), everolimus was expected to be the most effective treatment. Possibly this discrepancy can be explained by subclonal PTEN alterations resulting in differential efficacy of everolimus. Since this patient was not treated with either of these agents no correlation to the clinical response could be made, however it does show the additive value of drug screen on cancer organoids to genetic analysis of the tumor (58).

### Pulmonary Cancer

Lung cancer is the leading cause of cancer mortality and can be subdivided in non-small cell lung cancer (NSCLC) and small cell lung cancer (SCLC). The first lung cancer organoid biobank was established using 80 lung cancer (SCLC and NSCLC) patients. Drug sensitivity testing was performed for both cytotoxic drugs (docetaxel) and targeted agents (olaparib and erlotinib). Sensitivity to PARP inhibitor olaparib correlated with BRCA2 mutation status as expected. EGFR-targeting by erlotinib did correlate with EGFR mutation status in most, but not all, lung cancer organoids. In one patient that harbored an EGFR mutation but was resistant to erlotinib, a MET amplification was present explaining resistance (60). In another study, NSCLC organoids were derived from lung adenocarcinoma using a TP53 activator (nutlin) to eliminate normal lung stem cells and organoids were used as a potential drug screening model from which findings could be correlated to molecular markers. Lung cancer organoids with an ALK1 mutation were shown to be resistant to crizotinib whereas ERBB2 mutated cancer organoids were sensitive to erlotinib and gefitinib (59). Both studies did not compare the organoid response with that observed in the patient. Two more studies used NSCLC organoids for drug screening and correlation to molecular alterations found in the tumor. Both studies identified differential response towards treatment between organoids from different patients (61, 62) but also some treatments (such as vincristine) which showed comparable activity across all organoid lines (62).

### Head-and-Neck Cancer

Head-and-neck squamous cell carcinoma (HNSCC) organoids were developed and tested for treatment response towards standard-of-care chemotherapy in the metastasized setting (cisplatin and docetaxel). This study observed the highest IC50 value for docetaxel in the organoids derived from a pre-treated relapsed patient. Furthermore, *in vitro* resistance towards either one of the treatments could be confirmed in *in vivo* mouse models (63). HNSCC organoids were subjected to drug screening for targeted agents and standard-of-care treatments (cisplatin, carboplatin, cetuximab or radiotherapy) and differential responses were observed between organoids from different patients. Additionally, for a subset of patients, radiosensitivity of the organoids could be compared to clinical response. For six out of seven patients the organoid response towards radiation was similar to the clinical outcome of the patient (64). Another study used HNSCC organoids to test a novel treatment approach, EGFR-targeted photodynamic therapy. A patient-specific response was observed which correlated with EGFR levels exhibited in the tumor and corresponding organoids (65).

## Discussion

In the past decade, cancer organoids have been established for large set of solid tumors and extensively characterized on a genetic, transcriptomic and phenotypic level. Overall, the conclusion is that cancer organoids are genetically and phenotypically stable replicates of the tissue and tumor subtype characteristics. Multiple studies have also used cancer organoids for drug screening approaches. However, only few studies have been able to make a quantitative clinical comparison to derive predictive values (11, 23, 25, 51). Most studies to date still provide a descriptive comparison between the organoid and clinical response which highlights the potential value of cancer organoids for this utility. Nonetheless, larger cancer organoid studies that make a quantitative comparison to the clinical response are warranted to make accurate statements about the sensitivity and specificity of cancer organoids as clinical predictors of response and outcome. Cancer organoids have the potential to improve patient selection as multiple studies have shown that in some cases cancer organoids responded differently than predicted by driver mutations in the tumor (51, 57). This could for example be due to small tumor subclones or mutations downstream or parallel to the targeted pathway (60). Recently, a protocol has been published as a standardized method to successfully establish organoids from different cancer types and perform drug screening thereof (71). Such guidelines are crucial to develop a robust and reproducible co-clinical platform for cancer organoids.

### Limitations in Cancer Organoids Studies

Several limitations currently exist that need to be addressed before cancer organoids can be implemented as a co-clinical track to aid clinical decision making. First, the derivation of organoids is not equally effective for all solid cancers. For example, NSCLC has shown a low establishment rate due to frequent overgrowth of lung cancer organoids by normal airway cells (72). This overgrowth of somatic stem cells has been observed in the derivation of liver, prostate and endometrial cancer organoids as well (31, 43, 48). Approaches using omission of growth factors or addition of drugs based on molecular alterations of the tumor cells have been used to achieve pure cancer cell populations. However, these approaches are not universally applicable and vary greatly between cancer types but also between samples within the same cancer type (59, 73). Such approaches however should be avoided as they will reduce heterogeneity by eliminating subsets of tumor clones and may stimulate the outgrowth of others which will result in a reduced tumor representation and ability to predict treatment response. Future studies, including single-cell sequencing of organoids should be conducted to investigate how such counter selections affect tumor representation. Second, the derivation time of most cancer organoids is currently still weeks to months. If cancer organoids were to be used as co-clinical avatars this derivation time needs to be shortened to be of actual clinical value to the patient. Third, at the moment most patients die from metastatic disease. Heterogeneity between the primary tumor and developed metastases is an important cause of treatment failure in the metastasized setting. The opportunity to derive cancer organoids form different tumor sites provides the opportunity to select treatment options to which all distinct tumor locations share sensitivity. Multiple studies have successfully used this approach and showed differential as well as similar treatment responses for several anti-cancer agents between organoids derived from multiple tumor sites (33, 34, 51, 52). In clinical practice, taking multiple biopsies from one tumor or taking biopsies from multiple metastases might however not always be feasible which could potentially limit this application into the clinic.

Finally, the usage of exogenous growth factors and animal-derived basement membrane extracts (BME) in organoid cultures might influence reproducibility between organoid studies. There is currently no consensus on which medium components should be used, also not within one tumor type, which could explain differences found between studies with regards to treatment sensitivity. Moreover, the BMEs that are mostly used (i.e. Matrigel) feature variable compositions and the protein composition does not always reflect the stromal composition of the tissue the cancer cells were derived from. Utilizing a well-defined 3D matrix, adjusted to specific tumor types, might help to further improve cancer organoid studies (74).

### Incorporation of the Tumor Micro-environment in Cancer Organoids

Cells from the tumor micro-environment, such as stromal cells, immune cells and endothelial cells are lacking from cancer organoids. This may limit the utility of patient cancer organoids as predictors of treatment response as the tumor micro-environment is a key determinant of therapeutic outcome and a potential therapeutic target (75, 76). Importantly, hypoxia, a common characteristic of the tumor micro-environment of solid tumors which greatly contributes to malignant behavior and chemo- and radioresistance does develop in organoids once they reach a certain size (52). The importance of the tumor micro-environment has also been shown by incorporating cancer-associated fibroblasts in pancreatic cancer organoids which led to increased resistance towards treatment (68).

Several attempts have already been made towards incorporating aspects of the tumor micro-environment into the cancer organoid system (77). These include co-culture of tumor cells and immune cells or the preservation of original tumor micro-environmental components in the culture system (15). An example of this is the development of a co-culture system of peripheral blood mononuclear cells (PBMCs) and NSCLC or CRC cells. In this system, autologous tumor-reactive T-cells could be induced which were also shown to specifically kill tumor organoids whereas matched healthy airway organoids were unaffected (78). An air-liquid-interface (ALI) system was used to propagate cancer organoids directly from human or mouse tumor biopsies with preservation of the immune stroma and original tumor T-cell receptor spectrum and used to model immune checkpoint blockade therapy (79). In another example, glioblastoma biopsies were cut into small pieces without using a BME. This approach showed retention of immune and endothelial cells during culture and could be used to test for CAR-T cell treatment. However, whether these immune cells remain functional after prolonged culture is still uncertain (80).

Whereas endothelial cells can be preserved in acute slice culture systems and by starting a culture directly from a tumor biopsy without disturbing the tissue architecture, re-creating functional blood vessels requires a very different approach. Towards this goal, human vascularized brain organoids by co-culturing brain organoids and *in vitro* differentiated iPSCs towards endothelial cells were generated (81). Furthermore, tumor-on-a-chip models have been developed that include a microfluidic model to mimic vasculature and a running blood stream to further mimic the *in vivo* drug delivery situation (82).

### Drug Response Standardization in Cancer Organoid Studies

In order to fully understand the potential of cancer organoids as patient avatars important aspects need to be addressed. Most studies to date have used arbitrary drug dosages or titration curves. While this is a fairly common approach in pre-clinical research, it is recommended to use human equivalent dosages (i.e. measured drug concentrations in cancer tissue *in vivo*) in treatment experiments using cancer organoids. These differences in drug dose could lead to survival of cell populations *in vitro* that do not die *in vivo* or vice versa. Furthermore, since all studies use different drugs schedules and dosages, discrepancies of the predictive value of organoids between different studies can occur. Changes in drug concentration due to differences in diffusion of metabolites, *in vivo* drug metabolism and limited drug penetrance as a result of physiological barriers such as the blood-brain-barrier (BBB) can change the efficacy of a treatment option due to inadequate drug concentration in the cancer organoid. Microfluidics and an artificial BBB using organ on a chip technology may advance the field in this respect (82, 83). Furthermore, anti-cancer drugs are administered in specific treatment schedules in clinical practice. Especially concurrent treatment with multiple systemic agents and/or radiotherapy is still lacking in most cancer organoid studies whereas this is a common treatment strategy in daily clinical practice. Another important consideration that requires critical analysis are the treatment endpoint assays used in cancer organoid studies. It is well established that short term proliferation and viability/cell death (apoptosis, BrdU, ATP assays) are not predictive for long term survival and tumor control probability in response to radiotherapy and chemotherapy. Organoids are composed of heterogeneous cell population in which cancer stem cells have different responses to treatment and constitute only a minor fraction of the organoid population. Therefore, ninety percent cell death may not involve the most resistant clones within the organoid or the tumor in the patient. As tumor stem cells are intrinsically more resistant to treatment (84, 85) than bulk -non tumor stem cells–treatment schedules should also include long term survival assays (e.g. organoid replating studies, clonogenic assays) combined with tumor stem cell biomarkers to be more predictive for the tumor cure dose *in vivo*.

### Development of Recurrent Cancer Organoids

An interesting application of cancer organoids to be further explored is the development of organoids from recurrent cancer. Some studies describe organoids from biopsies from recurrent tumors, both with and without matched primary tumors (45, 47). Another interesting approach could be the *in vitro* development of a recurrent organoid from primary tumor organoids (10). Under selection of treatment pressure, resistant subpopulations within the tumor could potentially outgrow the treatment-sensitive cell populations or cause the acquisition of new mutations that cause resistance or may identify new therapeutic targets. Future studies should address if *in vitro* induced resistance mechanisms mimic the behavior of recurrent disease in patients.

### Corresponding Cancer and Healthy Tissue Organoids

Another important advantage of organoids is the possibility to simultaneously culture cancer and healthy tissue organoids from the same patient. Several studies have already implemented this technique in which it supported selection of the most promising treatment option (38, 50, 78, 86). Normal tissue toxicity is one of the main dose-limiting factors in cancer therapy, and being able to predict this can be of great benefit towards both optimal cancer treatment as well as maximizing quality of life. Thus, organoids are not only useful to individualize treatment in order to eradicate cancer cells but also to exclude potentially toxic treatments. The use of normal tissue organoids in parallel is only just emerging but would be a key feature to the armamentarium of organoids as clinical avatars for personalized precision medicine.

## Conclusion

In conclusion, cancer organoids exhibit the potential to act as predictors for clinical treatment response. However, quantitative data to make accurate statements about their predictive value is mainly lacking from current studies which is warranted to work towards their clinical implementation.

Biobanks of cancer organoids can be used to identify targetable mutations and patient subgroups to stratify patients for specific anti-cancer treatment options, beyond single predictive molecular markers. This approach is currently utilized by actively involving cancer organoids in ongoing clinical trials to improve patient selection (NCT03416244; NCT03307538).

Finally, cancer organoids can act as living surrogates (patient avatars) to use for high-throughput drug screening approaches to aid directly in the treatment of the specific patient that the organoid has been derived from and for the discovery of novel drug and targets. This possibility opens up major possibilities in the selection of personalized treatment options and the prevention of normal tissue toxicity.

## Author Contributions

MVe: conceptualization, data curation, investigation, writing (original draft, review, and editing), and final approval. AH: analysis and interpretation of data, writing (review), and final approval. DD: analysis and interpretation of data, writing (review), and final approval. MVo: conceptualization, data curation, investigation, supervision, writing (original draft, review, and editing), and final approval. All authors contributed to the article and approved the submitted version.

## Funding

KWF Kankerbestrijding (Dutch Cancer Society) Unique High Risk (16698/2018-1).

## Conflict of Interest

The authors declare that the research was conducted in the absence of any commercial or financial relationships that could be construed as a potential conflict of interest.

## References

[1] Sato T Vries RG Snippert HJ van de Wetering M Barker N Stange DE. Single Lgr5 stem cells build crypt-villus structures in vitro without a mesenchymal niche. Nature (2009) 459(7244):262–5. 10.1038/nature07935 19329995

[2] van de Wetering M Francies HE Francis JM Bounova G Iorio F Pronk A. Prospective derivation of a living organoid biobank of colorectal cancer patients. Cell (2015) 161(4):933–45. 10.1016/j.cell.2015.03.053 PMC6428276 25957691

[3] Roerink SF Sasaki N Lee-Six H Young MD Alexandrov LB Behjati S. Intra-tumour diversification in colorectal cancer at the single-cell level. Nature (2018) 556(7702):457–62. 10.1038/s41586-018-0024-3 29643510

[4] Clevers H. Modeling Development and Disease with Organoids. Cell (2016) 165(7):1586–97. 10.1016/j.cell.2016.05.082 27315476

[5] Weeber F Ooft SN Dijkstra KK Voest EE. Tumor Organoids as a Pre-clinical Cancer Model for Drug Discovery. Cell Chem Biol (2017) 24(9):1092–100. 10.1016/j.chembiol.2017.06.012 28757181

[6] Bleijs M van de Wetering M Clevers H Drost J. Xenograft and organoid model systems in cancer research. EMBO J (2019) 38(15):e101654. 10.15252/embj.2019101654 31282586 PMC6670015

[7] Drost J Clevers H. Organoids in cancer research. Nat Rev Cancer (2018) 18(7):407–18. 10.1038/s41568-018-0007-6 29692415

[8] Koppens MA Bounova G Cornelissen-Steijger P de Vries N Sansom OJ Wessels LF. Large variety in a panel of human colon cancer organoids in response to EZH2 inhibition. Oncotarget (2016) 7(43):69816–28. 10.18632/oncotarget.12002 PMC5342517 27634879

[9] Verissimo CS Overmeer RM Ponsioen B Drost J Mertens S Verlaan-Klink I. Targeting mutant RAS in patient-derived colorectal cancer organoids by combinatorial drug screening. Elife (2016) 5. 10.7554/eLife.18489 PMC5127645 27845624

[10] Buzzelli JN Ouaret D Brown G Allen PD Muschel RJ. Colorectal cancer liver metastases organoids retain characteristics of original tumor and acquire chemotherapy resistance. Stem Cell Res (2018) 27:109–20. 10.1016/j.scr.2018.01.016 PMC5842239 29414601

[11] Vlachogiannis G Hedayat S Vatsiou A Jamin Y Fernandez-Mateos J Khan K. Patient-derived organoids model treatment response of metastatic gastrointestinal cancers. Science (2018) 359(6378):920–6. 10.1126/science.aao2774 PMC6112415 29472484

[12] Gao M Lin M Rao M Thompson H Hirai K Choi M. Development of Patient-Derived Gastric Cancer Organoids from Endoscopic Biopsies and Surgical Tissues. Ann Surg Oncol (2018) 25(9):2767–75. 10.1245/s10434-018-6662-8 30003451

[13] Li X Francies HE Secrier M Perner J Miremadi A Galeano-Dalmau N. Organoid cultures recapitulate esophageal adenocarcinoma heterogeneity providing a model for clonality studies and precision therapeutics. Nat Commun (2018) 9(1):2983. 10.1038/s41467-018-05190-9 30061675 PMC6065407

[14] Yan HHN Siu HC Law S Ho SL Yue SSK Tsui WY. A Comprehensive Human Gastric Cancer Organoid Biobank Captures Tumor Subtype Heterogeneity and Enables Therapeutic Screening. Cell Stem Cell (2018) 23(6):882–97.e11. 10.1016/j.stem.2018.09.016 30344100

[15] Votanopoulos KI Mazzocchi A Sivakumar H Forsythe S Aleman J Levine EA. Appendiceal Cancer Patient-Specific Tumor Organoid Model for Predicting Chemotherapy Efficacy Prior to Initiation of Treatment: A Feasibility Study. Ann Surg Oncol (2019) 26(1):139–47. 10.1245/s10434-018-7008-2 PMC7992871 30414038

[16] Li J Xu H Zhang L Song L Feng D Peng X. Malignant ascites-derived organoid (MADO) cultures for gastric cancer in vitro modelling and drug screening. J Cancer Res Clin Oncol (2019) 145(11):2637–47. 10.1007/s00432-019-03004-z PMC11810372 31598791

[17] Schumacher D Andrieux G Boehnke K Keil M Silvestri A Silvestrov M. Heterogeneous pathway activation and drug response modelled in colorectal-tumor-derived 3D cultures. PloS Genet (2019) 15(3):e1008076. 10.1371/journal.pgen.1008076 30925167 PMC6457557

[18] Seidlitz T Merker SR Rothe A Zakrzewski F von Neubeck C Grutzmann K. Human gastric cancer modelling using organoids. Gut (2019) 68(2):207–17. 10.1136/gutjnl-2017-314549 PMC6352409 29703791

[19] Ubink I Bolhaqueiro ACF Elias SG Raats DAE Constantinides A Peters NA. Organoids from colorectal peritoneal metastases as a platform for improving hyperthermic intraperitoneal chemotherapy. Br J Surg (2019) 106(10):1404–14. 10.1002/bjs.11206 PMC6771632 31197820

[20] Pasch CA Favreau PF Yueh AE Babiarz CP Gillette AA Sharick JT. Patient-Derived Cancer Organoid Cultures to Predict Sensitivity to Chemotherapy and Radiation. Clin Cancer Res (2019) 25(17):5376–87. 10.1158/1078-0432.CCR-18-3590 PMC6726566 31175091

[21] Steele NG Chakrabarti J Wang J Biesiada J Holokai L Chang J. An Organoid-Based Preclinical Model of Human Gastric Cancer. Cell Mol Gastroenterol Hepatol (2019) 7(1):161–84. 10.1016/j.jcmgh.2018.09.008 PMC6279812 30522949

[22] Ganesh K Wu C O’Rourke KP Szeglin BC Zheng Y Sauve CG. A rectal cancer organoid platform to study individual responses to chemoradiation. Nat Med (2019) 25(10):1607–14. 10.1038/s41591-019-0584-2 PMC7385919 31591597

[23] Ooft SN Weeber F Dijkstra KK McLean CM Kaing S van Werkhoven E. Patient-derived organoids can predict response to chemotherapy in metastatic colorectal cancer patients. Sci Transl Med (2019) 11(513). 10.1126/scitranslmed.aay2574 31597751

[24] Costales-Carrera A Fernandez-Barral A Bustamante-Madrid P Guerra L Cantero R Barbachano A. Plocabulin Displays Strong Cytotoxic Activity in a Personalized Colon Cancer Patient-Derived 3D Organoid Assay. Mar Drugs (2019) 17(11). 10.3390/md17110648 PMC6891270 31752287

[25] Yao Y Xu X Yang L Zhu J Wan J Shen L. Patient-Derived Organoids Predict Chemoradiation Responses of Locally Advanced Rectal Cancer. Cell Stem Cell (2020) 26(1):17–26.e6. 10.1016/j.stem.2019.10.010 31761724

[26] Narasimhan V Wright JA Churchill M Wang T Rosati R Lannagan TR. Medium-throughput drug screening of patient-derived organoids from colorectal peritoneal metastases to direct personalized therapy. Clin Cancer Res (2020) 26:3662–71. 10.1158/1078-0432.CCR-20-0073 PMC8366292 32376656

[27] Zerp SF Bibi Z Verbrugge I Voest EE Verheij M. Enhancing radiation response by a second-generation TRAIL receptor agonist using a new in vitro organoid model system. Clin Transl Radiat Oncol (2020) 24:1–9. 10.1016/j.ctro.2020.05.012 32577539 PMC7303921

[28] Derouet MF Allen J Wilson GW Ng C Radulovich N Kalimuthu S. Towards personalized induction therapy for esophageal adenocarcinoma: organoids derived from endoscopic biopsy recapitulate the pre-treatment tumor. Sci Rep (2020) 10(1):14514. 10.1038/s41598-020-71589-4 32884042 PMC7471705

[29] Arena S Corti G Durinikova E Montone M Reilly NM Russo M. A Subset of Colorectal Cancers with Cross-Sensitivity to Olaparib and Oxaliplatin. Clin Cancer Res (2020) 26(6):1372–84. 10.1158/1078-0432.CCR-19-2409 31831554

[30] Huang L Holtzinger A Jagan I BeGora M Lohse I Ngai N. Ductal pancreatic cancer modeling and drug screening using human pluripotent stem cell- and patient-derived tumor organoids. Nat Med (2015) 21(11):1364–71. 10.1038/nm.3973 PMC4753163 26501191

[31] Broutier L Mastrogiovanni G Verstegen MM Francies HE Gavarro LM Bradshaw CR. Human primary liver cancer-derived organoid cultures for disease modeling and drug screening. Nat Med (2017) 23(12):1424–35. 10.1038/nm.4438 PMC5722201 29131160

[32] Nuciforo S Fofana I Matter MS Blumer T Calabrese D Boldanova T. Organoid Models of Human Liver Cancers Derived from Tumor Needle Biopsies. Cell Rep (2018) 24(5):1363–76. 10.1016/j.celrep.2018.07.001 PMC6088153 30067989

[33] Tiriac H Belleau P Engle DD Plenker D Deschenes A Somerville TDD. Organoid Profiling Identifies Common Responders to Chemotherapy in Pancreatic Cancer. Cancer Discovery (2018) 8(9):1112–29. 10.1158/1538-7445.PANCA19-C57 PMC6125219 29853643

[34] Li L Knutsdottir H Hui K Weiss MJ He J Philosophe B. Human primary liver cancer organoids reveal intratumor and interpatient drug response heterogeneity. JCI Insight (2019) 4(2). 10.1172/jci.insight.121490 PMC6413833 30674722

[35] Hennig A Wolf L Jahnke B Polster H Seidlitz T Werner K. CFTR Expression Analysis for Subtyping of Human Pancreatic Cancer Organoids. Stem Cell Derived Organ Hum Dis Develop (2019) 2019:1024614. 10.1155/2019/1024614 PMC6525827 31191661

[36] Bian B Juiz NA Gayet O Bigonnet M Brandone N Roques J. Front Oncol (2019) 9:475. 10.3389/fonc.2019.00475 PMC6560163 31231611

[37] Driehuis E van Hoeck A Moore K Kolders S Francies HE Gulersonmez MC. Pancreatic cancer organoids recapitulate disease and allow personalized drug screening. Proc Natl Acad Sci U S A (2019) 116:26580–90. 10.1073/pnas.1911273116 PMC6936689 31818951

[38] Ponz-Sarvise M Corbo V Tiriac H Engle DD Frese KK Oni TE. Identification of Resistance Pathways Specific to Malignancy Using Organoid Models of Pancreatic Cancer. Clin Cancer Res (2019) 25(22):6742–55. 10.1158/1078-0432.CCR-19-1398 PMC6858952 31492749

[39] Castven D Becker D Czauderna C Wilhelm D Andersen JB Strand S. Application of patient-derived liver cancer cells for phenotypic characterization and therapeutic target identification. Int J Cancer (2019) 144(11):2782–94. 10.1002/ijc.32026 30485423

[40] Sharick JT Walsh CM Sprackling CM Pasch CA Pham DL Esbona K. Metabolic Heterogeneity in Patient Tumor-Derived Organoids by Primary Site and Drug Treatment. Front Oncol (2020) 10:553. 10.3389/fonc.2020.00553 32500020 PMC7242740

[41] Seppala TT Zimmerman JW Sereni E Plenker D Suri R Rozich N. Patient-derived Organoid Pharmacotyping is a Clinically Tractable Strategy for Precision Medicine in Pancreatic Cancer. Ann Surg (2020) 272:427–35. 10.1097/SLA.0000000000004200 PMC7901605 32657929

[42] Saltsman JA Hammond WJ Narayan NJC Requena D Gehart H Lalazar G. A Human Organoid Model of Aggressive Hepatoblastoma for Disease Modeling and Drug Testing. Cancers (Basel) (2020) 12(9). 10.3390/cancers12092668 PMC7563272 32962010

[43] Gao D Vela I Sboner A Iaquinta PJ Karthaus WR Gopalan A. Organoid cultures derived from patients with advanced prostate cancer. Cell (2014) 159(1):176–87. 10.1016/j.cell.2014.08.016 PMC4237931 25201530

[44] Girda E Huang EC Leiserowitz GS Smith LH. The Use of Endometrial Cancer Patient-Derived Organoid Culture for Drug Sensitivity Testing Is Feasible. Int J Gynecol Cancer (2017) 27(8):1701–7. 10.1097/IGC.0000000000001061 PMC5627540 28683005

[45] Lee SH Hu W Matulay JT Silva MV Owczarek TB Kim K. Tumor Evolution and Drug Response in Patient-Derived Organoid Models of Bladder Cancer. Cell (2018) 173(2):515–28.e17. 10.1016/j.cell.2018.03.017 29625057 PMC5890941

[46] Puca L Bareja R Prandi D Shaw R Benelli M Karthaus WR. Patient derived organoids to model rare prostate cancer phenotypes. Nat Commun (2018) 9(1):2404. 10.1038/s41467-018-04495-z 29921838 PMC6008438

[47] Kopper O de Witte CJ Lohmussaar K Valle-Inclan JE Hami N Kester L. An organoid platform for ovarian cancer captures intra- and interpatient heterogeneity. Nat Med (2019) 25(5):838–49. 10.1038/s41591-019-0422-6 31011202

[48] Boretto M Maenhoudt N Luo X Hennes A Boeckx B Bui B. Patient-derived organoids from endometrial disease capture clinical heterogeneity and are amenable to drug screening. Nat Cell Biol (2019) 21(8):1041–51. 10.1038/s41556-019-0360-z 31371824

[49] Mullenders J de Jongh E Brousali A Roosen M Blom JPA Begthel H. Mouse and human urothelial cancer organoids: A tool for bladder cancer research. Proc Natl Acad Sci USA (2019) 116(10):4567–74. 10.1073/pnas.1803595116 PMC6410883 30787188

[50] Calandrini C Schutgens F Oka R Margaritis T Candelli T Mathijsen L. An organoid biobank for childhood kidney cancers that captures disease and tissue heterogeneity. Nat Commun (2020) 11(1):1310. 10.1038/s41467-020-15155-6 32161258 PMC7066173

[51] de Witte CJ Espejo Valle-Inclan J Hami N Lohmussaar K Kopper O Vreuls CPH. Patient-Derived Ovarian Cancer Organoids Mimic Clinical Response and Exhibit Heterogeneous Inter- and Intrapatient Drug Responses. Cell Rep (2020) 31(11):107762. 10.1016/j.celrep.2020.107762 32553164

[52] Hubert CG Rivera M Spangler LC Wu Q Mack SC Prager BC. A Three-Dimensional Organoid Culture System Derived from Human Glioblastomas Recapitulates the Hypoxic Gradients and Cancer Stem Cell Heterogeneity of Tumors Found In Vivo. Cancer Res (2016) 76(8):2465–77. 10.1158/0008-5472.CAN-15-2402 PMC4873351 26896279

[53] Saengwimol D Rojanaporn D Chaitankar V Chittavanich P Aroonroch R Boontawon T. A three-dimensional organoid model recapitulates tumorigenic aspects and drug responses of advanced human retinoblastoma. Sci Rep (2018) 8(1):15664. 10.1038/s41598-018-34037-y 30353124 PMC6199308

[54] Scognamiglio G De Chiara A Parafioriti A Armiraglio E Fazioli F Gallo M. Patient-derived organoids as a potential model to predict response to PD-1/PD-L1 checkpoint inhibitors. Br J Cancer (2019) 121(11):979–82. 10.1038/s41416-019-0616-1 PMC6889147 31666667

[55] Loong HH Wong AM Chan DT Cheung MS Chow C Ding X. Patient-derived tumor organoid predicts drugs response in glioblastoma: A step forward in personalized cancer therapy? J Clin Neurosci (2020) 78:400–2. 10.1016/j.jocn.2020.04.107 32340843

[56] Chadwick M Yang C Liu L Gamboa CM Jara K Lee H. Rapid Processing and Drug Evaluation in Glioblastoma Patient-Derived Organoid Models with 4D Bioprinted Arrays. iScience (2020) 23(8):101365. 10.1016/j.isci.2020.101365 32731171 PMC7393526

[57] Sachs N de Ligt J Kopper O Gogola E Bounova G Weeber F. A Living Biobank of Breast Cancer Organoids Captures Disease Heterogeneity. Cell (2018) 172(1-2):373–86.e10. 10.1016/j.cell.2017.11.010 29224780

[58] Li X Pan B Song X Li N Zhao D Li M. Breast cancer organoids from a patient with giant papillary carcinoma as a high-fidelity model. Cancer Cell Int (2020) 20:86. 10.1186/s12935-020-01171-5 32206037 PMC7079375

[59] Sachs N Papaspyropoulos A Zomer-van Ommen DD Heo I Bottinger L Klay D. Long-term expanding human airway organoids for disease modeling. EMBO J (2019) 38(4). 10.15252/embj.2018100300 PMC6376275 30643021

[60] Kim M Mun H Sung CO Cho EJ Jeon HJ Chun SM. Patient-derived lung cancer organoids as in vitro cancer models for therapeutic screening. Nat Commun (2019) 10(1):3991. 10.1038/s41467-019-11867-6 31488816 PMC6728380

[61] Chen JH Chu XP Zhang JT Nie Q Tang WF Su J. Genomic characteristics and drug screening among organoids derived from non-small cell lung cancer patients. Thorac Cancer (2020) 11(8):2279–90. 10.1111/1759-7714.13542 PMC7396373 32633046

[62] Li Z Qian Y Li W Liu L Yu L Liu X. Human Lung Adenocarcinoma-Derived Organoid Models for Drug Screening. iScience (2020) 23(8):101411. 10.1016/j.isci.2020.101411 32771979 PMC7415928

[63] Tanaka N Osman AA Takahashi Y Lindemann A Patel AA Zhao M. Head and neck cancer organoids established by modification of the CTOS method can be used to predict in vivo drug sensitivity. Oral Oncol (2018) 87:49–57. 10.1016/j.oraloncology.2018.10.018 30527243 PMC6294331

[64] Driehuis E Kolders S Spelier S Lohmussaar K Willems SM Devriese LA. Oral Mucosal Organoids as a Potential Platform for Personalized Cancer Therapy. Cancer Discovery (2019) 9(7):852–71. 10.1158/2159-8290.CD-18-1522 31053628

[65] Driehuis E Spelier S Beltran Hernandez I de Bree R MW S Clevers H. Patient-Derived Head and Neck Cancer Organoids Recapitulate EGFR Expression Levels of Respective Tissues and Are Responsive to EGFR- Targeted Photodynamic Therapy. J Clin Med (2019) 8(11). 10.3390/jcm8111880 PMC6912517 31694307

[66] Janakiraman H Zhu Y Becker SA Wang C Cross A Curl E. Modeling rectal cancer to advance neoadjuvant precision therapy. Int J Cancer (2020) 147(5):1405–18. 10.1002/ijc.32876 31989583

[67] Pauli C Hopkins BD Prandi D Shaw R Fedrizzi T Sboner A. Personalized In Vitro and In Vivo Cancer Models to Guide Precision Medicine. Cancer Discovery (2017) 7(5):462–77. 10.1158/2159-8290.CD-16-1154 PMC5413423 28331002

[68] Liu J Li P Wang L Li M Ge Z Noordam L. Cancer-Associated Fibroblasts Provide a Stromal Niche for Liver Cancer Organoids That Confers Trophic Effects and Therapy Resistance. Cell Mol Gastroenterol Hepatol (2020) 11:407–31. 10.1016/j.jcmgh.2020.09.003 PMC7788239 32932015

[69] Chung V McDonough S Philip PA Cardin D Wang-Gillam A Hui L. Effect of Selumetinib and MK-2206 vs Oxaliplatin and Fluorouracil in Patients With Metastatic Pancreatic Cancer After Prior Therapy: SWOG S1115 Study Randomized Clinical Trial. JAMA Oncol (2017) 3(4):516–22. 10.1001/jamaoncol.2016.5383 PMC5665683 27978579

[70] Beltran H Oromendia C Danila DC Montgomery B Hoimes C Szmulewitz RZ. A Phase II Trial of the Aurora Kinase A Inhibitor Alisertib for Patients with Castration-resistant and Neuroendocrine Prostate Cancer: Efficacy and Biomarkers. Clin Cancer Res (2019) 25(1):43–51. 10.1158/1078-0432.CCR-18-1912 30232224 PMC6320304

[71] Driehuis E Kretzschmar K Clevers H. Establishment of patient-derived cancer organoids for drug-screening applications. Nat Protoc (2020) 15:3380–409. 10.1038/s41596-021-00494-5 32929210

[72] Dijkstra KK Monkhorst K Schipper LJ Hartemink KJ Smit EF Kaing S. Challenges in Establishing Pure Lung Cancer Organoids Limit Their Utility for Personalized Medicine. Cell Rep (2020) 31(5):107588. 10.1016/j.celrep.2020.107588 32375033

[73] Wallaschek N Niklas C Pompaiah M Wiegering A Germer CT Kircher S. Establishing Pure Cancer Organoid Cultures: Identification, Selection and Verification of Cancer Phenotypes and Genotypes. J Mol Biol (2019) 431(15):2884–93. 10.1016/j.jmb.2019.05.031 31150736

[74] Gjorevski N Sachs N Manfrin A Giger S Bragina ME Ordonez-Moran P. Designer matrices for intestinal stem cell and organoid culture. Nature (2016) 539(7630):560–4. 10.1038/nature20168 27851739

[75] Junttila MR de Sauvage FJ. Influence of tumour micro-environment heterogeneity on therapeutic response. Nature (2013) 501(7467):346–54. 10.1038/nature12626 24048067

[76] Joyce JA. Therapeutic targeting of the tumor microenvironment. Cancer Cell (2005) 7(6):513–20. 10.1016/j.ccr.2005.05.024 15950901

[77] Bar-Ephraim YE Kretzschmar K Clevers H. Organoids in immunological research. Nat Rev Immunol (2020) 20(5):279–93. 10.1038/s41577-019-0248-y 31853049

[78] Dijkstra KK Cattaneo CM Weeber F Chalabi M van de Haar J Fanchi LF. Generation of Tumor-Reactive T Cells by Co-culture of Peripheral Blood Lymphocytes and Tumor Organoids. Cell (2018) 174(6):1586–98.e12. 10.1016/j.cell.2018.07.009 30100188 PMC6558289

[79] Neal JT Li X Zhu J Giangarra V Grzeskowiak CL Ju J. Organoid Modeling of the Tumor Immune Microenvironment. Cell (2018) 175(7):1972–88 e16. 10.1016/j.cell.2018.11.021 30550791 PMC6656687

[80] Jacob F Salinas RD Zhang DY Nguyen PTT Schnoll JG Wong SZH. A Patient-Derived Glioblastoma Organoid Model and Biobank Recapitulates Inter- and Intra-tumoral Heterogeneity. Cell (2020) 180(1):188–204 e22. 10.1016/j.cell.2019.11.036 31883794 PMC7556703

[81] Pham MT Pollock KM Rose MD Cary WA Stewart HR Zhou P. Generation of human vascularized brain organoids. Neuroreport (2018) 29(7):588–93. 10.1097/WNR.0000000000001014 PMC6476536 29570159

[82] Ayuso JM Virumbrales-Munoz M McMinn PH Rehman S Gomez I Karim MR. Tumor-on-a-chip: a microfluidic model to study cell response to environmental gradients. Lab Chip (2019) 19(20):3461–71. 10.1039/C9LC00270G PMC6785375 31506657

[83] Ahn SI Sei YJ Park HJ Kim J Ryu Y Choi JJ. Microengineered human blood-brain barrier platform for understanding nanoparticle transport mechanisms. Nat Commun (2020) 11(1):175. 10.1038/s41467-019-13896-7 31924752 PMC6954233

[84] Cojoc M Mabert K Muders MH Dubrovska A. A role for cancer stem cells in therapy resistance: cellular and molecular mechanisms. Semin Cancer Biol (2015) 31:16–27. 10.1016/j.semcancer.2014.06.004 24956577

[85] Baumann M Krause M Hill R. Exploring the role of cancer stem cells in radioresistance. Nat Rev Cancer (2008) 8(7):545–54. 10.1038/nrc2419 18511937

[86] Hou S Tiriac H Sridharan BP Scampavia L Madoux F Seldin J. Advanced Development of Primary Pancreatic Organoid Tumor Models for High-Throughput Phenotypic Drug Screening. SLAS Discovery (2018) 23(6):574–84. 10.1177/2472555218766842 PMC6013403 29673279

